# D-Dimer and Carcinoembryonic Antigen Levels: Useful Indicators for Predicting the Tumor Stage and Postoperative Survival

**DOI:** 10.1155/2016/4295029

**Published:** 2016-08-29

**Authors:** Kemal Tekeşin, Savaş Bayrak, Varol Esatoğlu, Ebru Özdemir, Leyla Özel, Veli Melih Kara

**Affiliations:** ^1^Department of General Surgery, Haydarpaşa Numune Training and Research Hospital, Istanbul, Turkey; ^2^Department of General Surgery, Istanbul Training and Research Hospital, Istanbul, Turkey

## Abstract

The purpose of this prospective study is to determine the preoperative plasma D-dimer and serum Carcinoembryonic Antigen (CEA) levels of patients scheduled for curative surgical resection for colorectal cancer and to evaluate the significance of these levels on the prognosis and postoperative survival rate. One hundred sixty-five patients with colorectal cancer, who were scheduled to have elective resection between January 2008 and January 2011, were included in the study. A significant increase was observed in the D-dimer levels, particularly in poorly differentiated tumors. The distance covered by the tumor inside the walls of the colon and rectum (T-stage) was significant for both D-dimer and CEA levels. As the T-stage increased, there was also a significant increase in the D-dimer and CEA levels. A high significance and correlation level was detected between the TNM staging and both D-dimer and CEA. A significant relationship was found between the advanced tumor stage and short postoperative survival rate of patients with colorectal cancer. Therefore, the analysis of preoperative D-dimer and CEA levels can be useful in predicting the stage and differentiation of the tumor and the postoperative survival rate.

## 1. Introduction

Studies about diagnosis, staging, and prognosis of colorectal cancer are still of interest for researchers. This study focuses on identifying the preoperative plasma D-dimer and serum Carcinoembryonic Antigen (CEA) levels of patients scheduled for curative surgical resection for colorectal cancer. Also it assesses the significance of D-Dimer and CEA levels on the prognosis and postoperative survival rate. The purpose of this prospective study is to determine the preoperative plasma D-dimer and serum Carcinoembryonic Antigen (CEA) levels of patients scheduled for curative surgical resection for colorectal cancer and to evaluate the significance of these levels on the prognosis and postoperative survival rate.

We investigated the relationship between the demographic features, tumor size, localization, and pathologic stage and the preoperative plasma D-dimer and serum CEA levels of patients as well as the contribution of these levels to survival during the postoperative follow-up.

## 2. Materials and Methods

One hundred sixty-five randomly selected patients with colorectal cancer who were scheduled for elective resection between January 2008 and January 2011 were included in the study. Informed consent was obtained from all the patients.

### 2.1. Patient Selection

For all patients, the arterial blood gas was measured and a venous Doppler ultrasound of the lower extremity was performed in order to identify patients with elevated D-dimer levels due to other conditions, such as deep vein thrombosis, pulmonary embolism, and coronary heart disease. Fourteen patients that had high D-dimer levels and were on antithromboembolic medicine due to pulmonary embolism and deep vein thrombosis or cardiac problems and 7 patients that were terminal due to early surgical complications (cardiopulmonary reasons and sepsis resulting from anastomosis leak) were excluded from the study. In addition, seven patients with unresectable liver metastasis and three patients with multiorgan involvement were also excluded from the study to eliminate the possible effect of their conditions on the postoperative survival rate. Finally, 134 patients who had elective resection were included in the study.

### 2.2. Examined Biochemical Parameters

In order to measure the plasma D-dimer and serum CEA levels, peripheral blood samples were collected after an overnight fasting three days before the operation day. DD was analyzed using the VIDAS® assay (Biomerieux®, USA). VIDAS D-Dimer Exclusion*™* II is an automated test for the immunoenzymatic determination of fibrin degradation products in human plasma.

CEA was measured using an electrochemiluminescence immunoassay with the Architect CEA® immunoassay analyzer (Abbott Laboratories).

The relationship between the demographic features, tumor size, localization, pathologic stage, and the preoperative plasma D-dimer and serum CEA levels of the patients was evaluated. In addition, the survival rate during the postoperative follow-up and the factors affecting the survival were examined.

### 2.3. Statistical Analysis

All data was coded and analyzed using the SPSS 16.0 (Statistical Package for the Social Sciences; SPSS, Chicago, Illinois, USA). The comparisons between the preoperative plasma D-dimer and serum CEA levels and between different patient groups were made using the nonparametric Mann–Whitney *U* test or Kruskal-Wallis test. The correlation of double tests including numeric variables was performed using the Spearman rank correlation test. Patients were followed up for survival analysis according to the follow-up protocol until January 2011. The median follow-up duration was 18 months. The results of this study were analyzed using the Kaplan-Meier survival curves (log rank tests) and the proportional hazard model. The values of *p* < 0.05 were accepted as statistically significant.

## 3. Results

Of the 134 patients, 50 were female and 84 were male. The median age was 62.5 (*r* = 31–84). The tumor localization was in colon in 94 of the patients and rectum in 40. Twelve patients with distal rectum tumor were operated on. In preoperative imaging, resectable liver metastasis was detected in 28 patients. Twenty-two of these patients underwent liver metastasectomy and/or radio frequency ablation (RF) and the remaining underwent minor/major hepatectomy with simultaneous colon resection. Tumor stages were evaluated according to the TNM staging system. Of the total 134 patients, 14 were grouped into stage 1, 34 into stage 2, 58 into stage 3, and 28 into stage 4. The median preoperative plasma D-dimer level was measured as 0.096 *μ*g/mL) and the serum CEA level was 3.96 (ng/mL). No correlation was detected between the age and the investigated parameters when the preoperative plasma D-dimer and serum CEA levels were evaluated. The D-dimer and CEA levels in blood were found to be similarly distributed by age. Similarly, no correlation was detected between sex and the investigated parameters when the preoperative plasma D-dimer and serum CEA levels were evaluated according to sex (*p* = 0.819 and *p* = 0.318, resp.).

Table 1 presents the relationship between the investigated parameters (tumor localization, size, and pathologic stage) and the plasma D-dimer and serum CEA levels. The Mann–Whitney *U* test, Kruskal-Wallis test, and Spearman's rank correlation tests were used to perform a statistical analysis of the correlation between the plasma D-dimer levels and CEA, lymph node, metastasis, and T-stage. The evaluation of the parameters showed no significant relationship between the localization of the tumor (colon/rectum) and the D-dimer and CEA levels. However, a statistical significance was observed between the differentiation of the tumor and D-dimer. It was also observed that the D-dimer levels were significantly elevated, particularly in poorly differentiated tumors (*p* = 0.04).

No significance was detected between the tumor size and the D-dimer and CEA levels. However, the distance that the tumor spread within the wall (T-stage) was found to be significant for both D-dimer and CEA levels. As the T-stage increased, the D-dimer and CEA levels also increased significantly. Similarly, a high significance and correlation was detected between the TNM stage and both the D-dimer and CEA levels.

### 3.1. Postoperative Follow-Up

Except for six patients who had been diagnosed at an early stage after the operation, all the remaining patients (128) were treated with 5-FU-based (leucovorin/oxaliplatin) adjuvant chemotherapy protocol. The median duration of follow-up was 18 ± 0.5 months (*r* = 4–31). During the term of the study, new metastases were detected in 16 patients (liver only 9, lung and liver 5, and brain 2). Thirty patients (22%) became terminal.

**Figure 1:**
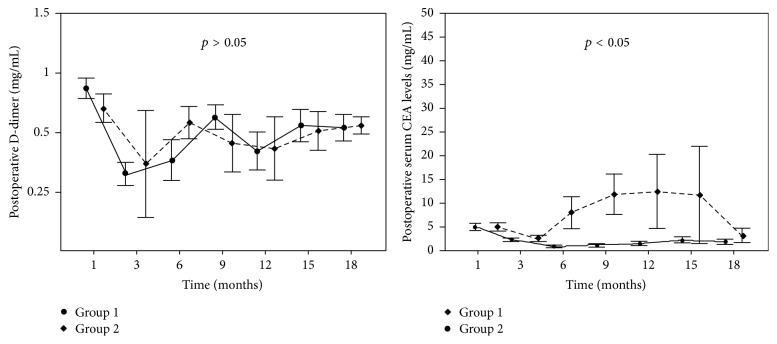
Relationship between metastasis and postoperative D-dimer and CEA levels of patients.

The comparison of the D-dimer levels of the groups showed no significant relationship between metastasis and the postoperative D-dimer levels (*p* > 0.05). However, a significant relation between metastasis and postoperative CEA levels was found when the serum CEA levels of the groups were compared (*p* < 0.05) (Figure 1).

**Figure 2:**
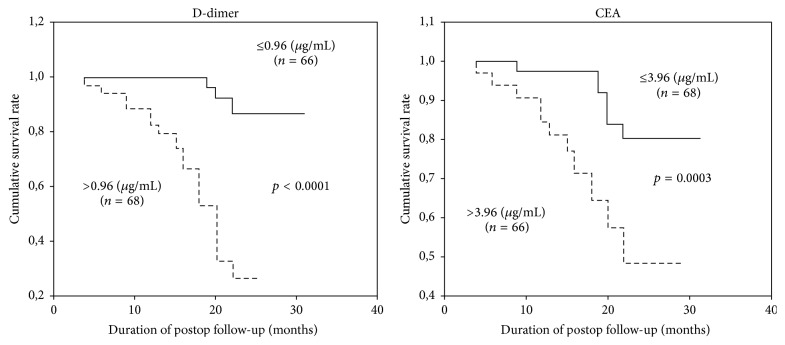
Relationship between the postoperative general survival and preoperative D-dimer and CEA levels.

Figure 2 presents the relationship between the postoperative general survival and preoperatively measured D-dimer and CEA levels. A strong correlation was observed between the general survival and preoperative median D-dimer and CEA levels of the patients (Kaplan-Meier results: *p* = 0.0001 for D-dimer and *p* = 0.0003 for CEA). The patients with higher preoperative D-dimer and CEA levels had shorter survival time, which indicated that high values had an impact on the survival.

The evaluation of the prognostic significance of the parameters using the univariate variance analysis (Table 2) showed that the depths of the wall invasion and lymph node invasion, advanced tumor stage, high plasma D-dimer, and serum CEA levels resulted in a significantly shorter survival time.

A multivariate analysis was performed using the significant data from the univariate variance analysis.  Table 3 presents the results of the multivariate analysis. These results show that the depths of the wall and lymph node invasion, metastasis, advanced tumor stage, high plasma D-dimer, and serum CEA levels caused a significantly shorter survival time.

## 4. Discussion

Solid tumor formation depends on the proliferative activity and angiogenesis of the tumor. It has been reported that, at the first stage of tumor development, avascular neoplastic cell groups can grow up to 2-3 mm in diameter and maintain their cellular functions via diffusion until the 106-cell stage [1–4]. However, when tumor cells reach the threshold limit and size and can no longer feed via diffusion, those with an increased need for nutrients and oxygen and the deoxygenated cells secrete angiogenic factors and cause new capillary formation [5–7]. For the tumor cell to develop, grow, and make local invasion and far metastasis, new vessel and capillary webs are needed to be formed [5–7]. A tumor cell meets these requirements using the specific growth factors it secretes and obtains from the surrounding immune cells, like fibroblasts, macrophages, and other cells. Many factors, particularly VEGF and FGF, play an important role in the formation of a new vascular structure. These specific growth factors cause the endothelial cells to proliferate by activating the endothelial cell receptors, thus resulting in new vessel formation.

There are basic differences distinguishing tumor vessels from normal vessels. Endothelial cells constituting the tumor capillary are bigger and there are more open spaces between these cells [8]. In addition, the basal membrane of the tumor capillary produces a thinner layer compared to the normal vessels. These differences explain the increase in microvascular permeability and the pathophysiologic abnormalities, which results in the ability for metastasis [8].

The vessel structure becomes weak and fragile under pressure and the abnormal blood flow causes hypercoagulation [9]. Increased transcapillary permeability allows the tumor cells that are bigger than normal blood cells to pass from the intravascular space and set the ground for tumor emboli. This pathophysiologic process causes the coagulation cascade to be activated more than normal [9].

Even though literature presents a number of studies that explain mechanism of the hypercoagulability in patients with colorectal cancer, a clear explanation is still missing. For instance, Wojtukiewicz et al. pointed out that coagulation is activated by soluble products at distant from the tumor [10]. Dover et al. found significantly more factor X-activating activity in colorectal tumor tissue than in the normal mucosa [11]. Pineo et al. detected that mucin has a role in hypercoagulation but this does not explain the origin of mucin [12]. It should be concluded that further biochemical and histopathological research is required to find the reason of hypercoagulability in patients with colorectal cancer.

In addition, immune cells, such as fibroblast and macrophage surrounding the tumor cells, constitute the extracellular matrix of the tumor. Fibroblasts in this matrix lead to neoangiogenesis and the formation of cross-linked fibrin in the extracellular matrix by secreting specific growth factors [13, 14]. There is a continuous cycle of formation and degradation of fibrin matrix and this cycle causes fibrin degradation products, D-dimer, to increase [14–16].

The invasive and metastatic ability of tumor cells is dependent on the formation of the fibrin matrix and the enlargement of the vascular web. As the tumor cell grows and the fibrin matrix and the vascular web are formed, hypercoagulation continues to increase [14, 15]. These two pathophysiologic processes lead to the activation of coagulation cascade and an increase in fibrin degradation products in the circulation. As a result, D-dimer, as one of the fibrin degradation products, also increases.

D-dimer is an important marker used clinically since it is elevated in hemostatic disorders, such as disseminate intravascular coagulation, deep vein thrombosis, pulmonary embolism, and coronary heart disease. Studies have shown that 90% of cancer patients have hemostatic disorders, shortened prothrombin and partial thromboplastin time, and increased factors II, V, VIII, IX, XI, and XII, fibrinogen, and fibrin degradation products [14]. The preoperative D-dimer levels have been reported to increase in relation to the stage of the tumor in patients with prostate, lung, cervix, ovary, breast, or colorectal cancer [9, 17–26].

In the current study, the preoperative CEA and D-dimer levels were found related to the tumor stage in patients with colorectal cancer. Since the invasive and metastatic ability of tumors depends on the formation of new vessels, increased fibrin turnover, and a resulting increase in the fibrin degradation products, the D-dimer levels were measured to be high in advanced stage tumors.

In our study, the preoperative CEA and D-dimer levels were significantly elevated in patients with T-stage, lymph node positivity, and distant metastasis. This is more distinct in the case of metastasis, which can be attributed to the increased angiogenesis in the primary tumor as well as the tumor emboli in the circulation. There is a strong relationship between the preoperative D-dimer levels and tumor differentiation. In our study, the D-dimer levels were significantly elevated in aggressive and poorly differentiated tumors with a high ability of invasion. This research demonstrates that changes in D-dimer and CEA levels depend on feature of the tumor. This finding is consistent with results of the previous studies [27–30].

D-dimer and CEA levels may also be used as indicators for monitoring response of patients during anti-VEGF therapy. Literature shows a number of studies that validate this argument. For instance, Blackwell et al. demonstrated that elevated D-dimer levels were correlated significantly with increased mortality in patient who has taken combined chemotherapy and anti-VEGF therapy. This study also shows that elevations in D-dimer values have a stronger correlation with disease progression than did elevations in CEA.

Similar results were found in a research carried out by Kilic et al. for 51 all-stage CRC patients who underwent resection [31, 32].

In the univariate and multivariate analyses where patients with high preoperative plasma D-dimer levels were compared to those with low levels, a strong correlation was detected in the postoperative survival. Similarly, Blackwell et al. and Oya et al. found a strong correlation between the preoperative D-dimer levels and postoperative survival [31, 33]. In the current study, the postoperative D-dimer levels were also found to recover to normal levels in the postoperative 3rd month, increase with chemotherapy, and then fluctuate. In addition, the postoperative D-dimer levels do not provide any information about the possibility of metastasis or survival. This may be led by the limited follow-up period of our study (18 months). However, a significant relationship was observed between the high serum CEA levels and metastasis in the postoperative follow-up.

## 5. Conclusions

The preoperative plasma D-dimer levels are significantly elevated in advanced stage tumors with deep wall invasion, lymph node involvement, metastasis, and poor differentiation. In addition, there is a significant relationship between advanced tumor stage and short postoperative survival and high preoperative D-dimer and CEA levels in patients with colorectal cancer. In the light of these results, we conclude that the preoperative D-dimer and CEA levels can be useful in differential diagnosis and predicting the tumor stage and postoperative survival. Therefore, it is also concluded that clinician should obtain D-dimer and CEA levels as part of routine care.

This research has contributed the literature that aims to clarify the situation of hypercoagulation in colorectal cancer. Further studies should examine other proteins such as fibrinogen and plasminogen within coagulation system. Thus, therapy that targets the host environment might be developed for patients with colorectal cancers.

## Figures and Tables

**Table 1:** Relationship between the investigated parameters and the plasma D-dimer and serum CEA levels.

	*n* = 134	D-dimer		CEA	
		Median (ng/mL)	*p*_D-dimer_	Median (ng/mg)	*p*_CEA_
Tumor localization					
Colon	94	1.10	0.472^*∗*^	26.1	0.621^*∗*^
Rectum	40	0.96		26.9	
Differentiation					
Well	18	1.10	0.04^*α*^	26.8	0.207^*α*^
Middle	94	1.15		27.3	
Poor/mucinous	22	1.80		33.1	
Tumor diameter (mm)					
<20 mm	6	1.31	0.276^*α*^	2.8	0.615^*α*^
20–50 mm	86	1.25		23.5	
>50 mm	42	1.28		36.6	
Wall invasion (N0, M0)					
T1	5	0.14	0.0001^+^	2.9	0.0001^+^
T2	16	0.39		3.7	
T3	70	0.80		15.6	
T4	18	1.30		50.5	
Lymph node (any T, M0)					
Negative	52	0.55	0.0004^*∗*^	4.8	0.02^*∗*^
Positive	54	0.96		40.8	
Metastasis (any T and N)					
No	106	0.78	>0.001^*∗*^	3.20	>0.001^*∗*^
Yes	28	2.53		4.80	
Stage					
1	14	0.30	0.001^+^	3.20	0.001^+^
2	34	0.66		4.80	
3	58	0.98		12.5	
4	28	2.53		102.7	

^*∗*^Mann–Whitney *U* test; ^*α*^Kruskal-Wallis test; ^+^Spearman's rank correlation test.

**Table 2:** Evaluation of the prognostic significance of the parameters using the univariate analysis of variance.

	HR	95% CI	*p*
Age	0.4629	0.2219–0.9657	0.168
Sex (female/male)	1.0748	0.5016–2.3032	0.852
Tumor size	1.6839	0.8512–3.3310	0.134
Tumor localization (colon/rectum)	0.4190	0.1707–1.0285	0.057
Differentiation (well/other)	0.0413	0.4811–2.2542	0.918
Wall invasion (T)	2.3222	1.1121–4.8501	0.02
Lymph node invasion (yes/no)	0.1312	0.0304–0.5661	0.006
Metastasis (yes/no)	6.4082	2.5226–16.2787	0.0001
Stage	3.6337	1.9390–6.8095	0.0001
D-dimer	2.1274	1.6926–2.6730	<0.0001
CEA	1.0008	1.0014–1.0109	0.01

HR: hazard ratio; CI: confidence interval.

**Table 3:** Multivariate analysis of the significant variables determined by univariate analysis.

	HR	95% CI	*p*
Wall invasion (T)	2.372	0.981–5.762	0.041
Lymph node invasion (N)	4.053	1.853–8.834	0.012
Metastasis (M)	3.325	1.883–8.632	0.023
D-dimer	1.873	1.032–3.494	0.021
CEA	4.084	1.262–11.873	0.053
*p*	0.0003		
Wall invasion (T)	2.074	1.023–4.376	0.044
Lymph node invasion (N)	3.763	1.912–8.352	0.013
Metastasis (M)	3.671	1.871–9.453	0.021
D-dimer	2.456	1.913–3.379	0.012
*p*	0.0001		
Wall invasion (T)	2.943	0.914–4.729	0.023
Lymph node invasion (N)	3.992	1.899–8.400	0.011
Metastasis (M)	6.783	1.711–8.213	0.032
CEA	4.112	1.057–10.964	0.044
*p*	0.02		
Lymph node invasion (N)	3.811	1.792–7.897	0.019
Metastasis (M)	4.011	1.989–6.113	0.035
D-dimer	1.879	0.942–3.435	0.012
CEA	3.353	1.126–13.632	0.037
*p*	0.0002		
Wall invasion (T)	2.341	1.247–6.983	0.034
Metastasis (M)	3.991	2.035–5.878	0.030
D-dimer	1.973	1.286–4.562	0.018
CEA	3.674	1.233–9.120	0.021
*p*	0.0001		
Wall invasion (T)	2.171	1.462–8.354	0.039
Lymph node invasion (N)	3.915	1.660–8.942	0.020
D-dimer	1.895	1.114–4.087	0.013
CEA	3.442	1.193–8.732	0.032
*p*	0.0002		

HR: hazard ratio; CI: confidence interval.

## References

[1] Folkman J. (1990). What is the evidence that tumors are angiogenesis dependent?. *Journal of the National Cancer Institute*.

[2] Weidner N., Semple J. P., Welch W. R., Folkman J. (1991). Tumor angiogenesis and metastasis—correlation in invasive breast carcinoma. *The New England Journal of Medicine*.

[3] Folkman J., Cole P., Zimmerman S. (1966). Tumor behavior in isolated perfused organs: in vitro growth and metastases of biopsy material in rabbit thyroid and canine intestinal segment. *Annals of Surgery*.

[4] Tannock I. F. (1970). Population kinetics of carcinoma cells, capillary endothelial cells, and fibroblasts in a transplanted mouse mammary tumor. *Cancer Research*.

[5] Shweiki D., Itin A., Soffer D., Keshet E. (1992). Vascular endothelial growth factor induced by hypoxia may mediate hypoxia-initiated angiogenesis. *Nature*.

[6] Thompson W. D., Shiach K. J., Fraser R. A., McIntosh L. C., Simpson J. G. (1987). Tumours acquire their vasculature by vessel incorporation, not vessel ingrowth. *The Journal of Pathology*.

[7] Ziche M., Gullino P. M. (1982). Angiogenesis and neoplastic progression in vitro. *Journal of the National Cancer Institute*.

[8] Blood C. H., Zetter B. R. (1990). Tumor interactions with the vasculature: angiogenesis and tumor metastasis. *Biochimica et Biophysica Acta (BBA)—Reviews on Cancer*.

[9] Buccheri G., Ferrigno D., Ginardi C., Zuliani C. (1997). Haemostatic abnormalities in lung cancer: prognostic implications. *European Journal of Cancer*.

[10] Wojtukiewicz M. Z., Zacharski L. R., Memoli V. A. (1989). Indirect activation of blood coagulation in colon cancer. *Thrombosis and Haemostasis*.

[11] Dover R., Goeting N. L. M., Taylor I., Roath O. S., Francis J. L. (1987). Factor X-activating activity in patients with colorectal carcinoma. *British Journal of Surgery*.

[12] Pineo G. F., Regoeczi E., Hatton M. W. C., Brain M. C. (1973). The activation of coagulation by extracts of mucus: a possible pathway of intravascular coagulation accompanying adenocarcinomas. *Journal of Laboratory and Clinical Medicine*.

[13] Kempin S. J. (1997). Hemostatic defects in cancer patients. *Cancer Investigation*.

[14] Lip G. Y., Chin B. S., Blann A. D. (2002). Cancer and the prothrombotic state. *The Lancet Oncology*.

[15] Gouin-Thibault I., Samama M. M. (1999). Laboratory diagnosis of the thrombophilic state in cancer patients. *Seminars in Thrombosis and Hemostasis*.

[16] Verheul H. M. W., Hoekman K., Lupu F. (2000). Platelet and coagulation activation with vascular endothelial growth factor generation in soft tissue sarcomas. *Clinical Cancer Research*.

[17] Cooper D. L., Sandler A. B., Wilson L. D., Duffy T. P. (1992). Disseminated intravascular coagulation and excessive fibrinolysis in a patient with metastatic prostate cancer: response to epsilon-aminocaproic acid. *Cancer*.

[18] Geenen R. W. F., Delaere K. P. J., Van Wersch J. W. J. (1997). Coagulation and fibrinolysis activation markers in prostatic carcinoma patients. *European Journal of Clinical Chemistry and Clinical Biochemistry*.

[19] Wojtukiewicz M. Z., Zacharski L. R., Moritz T. E., Hur K., Edwards R. L., Rickles F. R. (1992). Prognostic significance of blood coagulation tests in carcinoma of the lung and colon. *Blood Coagulation and Fibrinolysis*.

[20] van Duijnhoven E. M., Lustermans F. A. T., Van Wersch J. W. J. (1993). Evaluation of the coagulation/fibrinolysis balance in patients with colorectal cancer. *Haemostasis*.

[21] Gabazza E. C., Taguchi O., Yamakami T., Machishi M., Ibata H., Suzuki S. (1993). Evaluating prethrombotic state in lung cancer using molecular markers. *Chest*.

[22] Taguchi O., Gabazza E. C., Yasui H., Kobayashi T., Yoshida M., Kobayashi H. (1997). Prognostic significance of plasma D-dimer levels in patients with lung cancer. *Thorax*.

[23] Gadducci A., Baicchi U., Marrai R. (1993). Pretreatment plasma levels of fibrinopeptide-A (FPA), D-dimer (DD), and von Willebrand Factor (vWF) in patients with operable cervical cancer: influence of surgical-pathological stage, tumor size, histologic type, and lymph node status. *Gynecologic Oncology*.

[24] von Tempelhoff G.-F., Dietrich M., Niemann F., Schneider D., Hommel G., Heilmann L. (1997). Blood coagulation and thrombosis in patients with ovarian malignancy. *Thrombosis and Haemostasis*.

[25] Rella C., Coviello M., De Frenza N. (1993). Plasma D-dimer measurement as a marker of gynecologic tumors: comparison with Ca 125. *Tumori*.

[26] Blackwell K., Haroon Z., Broadwater G. (2000). Plasma D-dimer levels in operable breast cancer patients correlate with clinical stage and axillary lymph node status. *Journal of Clinical Oncology*.

[27] Oya M., Akiyama Y., Yanagida T., Akao S., Ishikawa H. (1998). Plasma D-dimer level in patients with colorectal cancer: its role as a tumor marker. *Surgery Today*.

[28] Wanebo H. J., Rao B., Pinsky C. M. (1978). Preoperative carcinoembryonic antigen level as a prognostic indicator in colorectal cancer. *The New England Journal of Medicine*.

[29] Goslin R., Steele G., MacIntyre J. (1980). The use of preoperative plasma CEA levels for the stratification of patients after curative resection of colorectal cancers. *Annals of Surgery*.

[30] Moertel C. G., O'Fallon J. R., Go V. L. W., O'Connell M. J., Thynne G. S. (1986). The preoperative carcinoembryonic antigen test in the diagnosis, staging, and prognosis of colorectal cancer. *Cancer*.

[31] Blackwell K., Hurwitz H., Liebérman G. (2004). Circulating D-dimer levels are better predictors of overall survival and disease progression than carcinoembryonic antigen levels in patients with metastatic colorectal carcinoma. *Cancer*.

[32] Kilic M., Yoldas O., Keskek M. (2008). Prognostic value of plasma D-dimer levels in patients with colorectal cancer. *Colorectal Disease*.

[33] Oya M., Akiyama Y., Okuyama T., Ishikawa H. (2001). High preoperative plasma D-dimer level is associated with advanced tumor stage and short survival after curative resection in patients with colorectal cancer. *Japanese Journal of Clinical Oncology*.

